# MHC Class II Expression in Human Basophils: Induction and Lack of Functional Significance

**DOI:** 10.1371/journal.pone.0081777

**Published:** 2013-12-09

**Authors:** Astrid L. Voskamp, Sara R. Prickett, Fabienne Mackay, Jennifer M. Rolland, Robyn E. O'Hehir

**Affiliations:** 1 Department of Immunology, Monash University, Melbourne, Victoria, Australia; 2 Department of Allergy, Immunology and Respiratory Medicine, The Alfred Hospital and Monash University, Melbourne, Victoria, Australia; French National Centre for Scientific Research, France

## Abstract

The antigen-presenting abilities of basophils and their role in initiating a Th2 phenotype is a topic of current controversy. We aimed to determine whether human basophils can be induced to express MHC Class II and act as antigen presenting cells for T cell stimulation. Isolated human basophils were exposed to a panel of cytokines and TLR-ligands and assessed for MHC Class II expression. MHC Class II was expressed in up to 17% of isolated basophils following incubation with a combination of IL-3, IFN-γ and GM-CSF for 72 hours. Costimulatory molecules (CD80 and CD86) were expressed at very low levels after stimulation. Gene expression analysis of MHC Class II-positive basophils confirmed up-regulation of HLA-DR, HLA-DM, CD74 and Cathepsin S. However, MHC Class II expressing basophils were incapable of inducing antigen-specific T cell activation or proliferation. This is the first report of significant cytokine-induced MHC Class II up-regulation, at both RNA and protein level, in isolated human basophils. By testing stimulation with relevant T cell epitope peptide as well as whole antigen, the failure of MHC Class II expressing basophils to induce T cell response was shown not to be solely due to inefficient antigen uptake and/or processing.

## Introduction

Basophils are circulating effector cells involved in protection against helminth infection, by producing histamine and other inflammatory mediators upon activation by helminth-derived antigens [1]–[3]. By a similar mechanism, they also play a key role in eliciting allergic reactions to normally innocuous environmental antigens. In addition, basophils are significant producers of IL-4 [4]–[5] and IL-13 [6]–[7] and can induce T cell-independent IgE class switching in B cells [8]–[9].

In 2009, there were several reports from murine studies that basophils play a direct role in the initiation of a Th2 type response to particular antigens through antigen presentation [10]–[12]. This suggested a new paradigm for induction of Th2 response and the potential importance of basophils in initiating the development of allergic disorders. However, in 2010, these findings were contradicted, implicating a discrepancy in the basophil population depletion methods of the previous studies [13]–[14]. At this time a transgenic mouse model with constitutive and selective deletion of basophils was developed. Using this model, basophils were shown to be important for protective immunity against helminths, for the development of chronic allergic inflammation, but dispensable for the initiation of a primary Th2 response to antigens [15]. However, a more recent study using basophil conditionally-depleted transgenic mice showed that basophils can induce Th2 skewing to haptens and peptide antigens, but not protein antigens *in vivo*. In this model, basophils were shown to express MHC Class II and costimulatory molecules and could present exogenous peptide, but they were unable to take up and process whole antigen [16]. Therefore, the full extent of the role of the basophil in antigen presentation and Th2 development is as yet unclear.

Basophils comprise less than 1% of circulating leukocytes in humans and are generally considered to be HLA-DR negative. Therefore this marker is often used to distinguish basophils from circulating CD123^+^ plasmacytoid dendritic cells (pDC). Freshly isolated basophils from PBMC of healthy individuals have consistently been found to lack MHC Class II molecules regardless of the isolation method used [17]–[19]. Short-term stimulation (24 hours or less) of isolated basophils with allergens, anti-IgE or the TLR 2 ligand peptidoglycan did not induce HLA-DR expression [19] and only “marginal” levels were detected after stimulation with IL-3 and IFN-γ [17]–[18]. Accordingly, stimulated basophils were unable to induce T cell activation through presentation of whole antigen in these studies. Whether there are conditions under which these properties can be induced in human basophils has not yet been reported, nor has the ability of human basophils to present peptide to T cells, as recently shown in mice [16].

Evidence of HLA-DR expressing basophils in humans has been found in relation to disease. One study reported increased expression of HLA-DR on circulating basophils and basophil migration to lymph nodes and spleen of patients suffering from Systemic Lupus Erythematosus, but not healthy controls [20]. Basophils have also been detected in mucosal and other tissues and during inflammation in Th2 [21]–[22] and Th17 [23] associated diseases, revealing their ability to migrate from the circulation to areas where they may be exposed to MHC Class II inducing factors. It is therefore feasible that mature, circulating human basophils could be induced to express significant levels of MHC Class II under certain conditions, which may have relevance to other specific physiological conditions or disease states.

Although basophils share many similar cellular features with mast cells, human basophils may have a closer lineage relationship with eosinophils [24]–[25]. Previous *in vitro* studies showed MHC Class II expression and antigen presenting functions in eosinophils upon stimulation with specific cytokines. Maximal MHC Class II expression levels occurred at two to four days of stimulation [26]–[31], durations longer than examined in prior basophil studies. In order to ascertain the potential for mature human basophils to express MHC Class II and participate in antigen presentation, we chose to follow the methods used in these studies, including stimulation with IL-3, IFN-γ, GM-CSF and TLR ligands for up to 72 hours. In this report we assess the induction of MHC Class II and costimulatory molecule expression in mature human basophils and their ability to present antigen to induce specific T cell proliferation. As antigen, we chose a clinically relevant house dust mite (HDM) allergen extract and we compared T cell responses of Th2-predisposed HDM-allergic subjects with non-atopic subjects. As a tool to dissect antigen processing and presenting capability of activated basophils, we also tested peptide presentation to peptide-specific T cells.

## Materials and Methods

### Subjects

Four non-atopic and five atopic, HDM-allergic Caucasian adults were recruited through The Alfred Allergy Clinic, Melbourne, Australia. Allergic subjects had clinical symptoms of HDM allergy, HDM-specific IgE CAP score ≥2 (Pharmacia Diagnostics, Uppsala, Sweden) and skin prick test wheals >4 mm diameter to HDM extract. Non-atopic subjects had no history of clinical symptoms of allergy and were skin prick test or IgE CAP score negative to a range of common allergens including HDM. The study was approved by The Alfred Hospital Ethics Committee (Project number 509/11) and the Monash University Human Research Ethics Committee (CF12/0702), and informed written consent was obtained from all subjects.

### Antigens and other stimulants

Lyophilised HDM extract was obtained from Greer Laboratories (NC, USA), reconstituted with PBS pH 7.2 and filter sterilized (0.2 µm) before use. The extract was confirmed to be neither mitogenic nor toxic as described [32] and the endotoxin level was 179 EU/mg (Endpoint Chromogenic LAL assay; Lonza, Walkersville, USA). Lyophilized *Arachis hypogaea* 1 (Ara h 1) peptide (ALMLPHFN) was synthesized by Mimotopes (Victoria, Australia) and reconstituted in 10% dimethyl sulfoxide/PBS. Lyophilized cytokines rhIL-3 (R&D Systems, Minneapolis, USA), rhIFN-γ, rhGM-CSF and rhTNF-α (MiltenyiBiotech, BergischGladbach, Germany) and TLR 4, 2 and 9 ligands LPS (Sigma-Aldrich, St Louis, MO), PGN and CpG (Invivogen, San Diego, USA) respectively were reconstituted in 0.1% BSA/PBS pH 7.2 according to manufacturer's recommendations.

### Basophil isolation, purity and culture

Basophils were isolated from ficoll-purified PBMC with a Human Basophil Enrichment kit (StemCell Technologies, Vancouver, Canada) according to the manufacturer's protocol (removing cells expressing CD2, CD3, CD14, CD15, CD19, CD24, CD34, CD36, CD45RA, CD56 and glycophorin A). Basophil purity was assessed with a combination of anti-IgE-FITC (Invitrogen, Carlsbad, USA), anti-CD123-PE (BD Pharmingen, San Diego, USA), anti-CD203c-APC (Miltenyi Biotech, Germany), anti-FcεRI-PE, anti-CD19-eF450, anti-CD4-APC eF780 (eBioscience, San Diego, USA) and, when sufficient cells were available, anti-Lineage-1-FITC (containing antibodies specific for CD3, CD19, CD14 and CD56; BD Pharmingen). Following isolation, basophils were stimulated with cytokines, TLR ligands or HDM in 96-U-well plates at 2×10^5^ cells/ml in 10% FCS RPMI consisting of RPMI-1640 containing 2 mM L-glutamine, 100 IU/mL penicillin-streptomycin and 10% FCS (Sigma-Aldrich, St Louis, USA) for 72 hours. Annexin V-PE (eBioscience) and 7AAD (BD Pharmingen) were used according to the manufacturers' protocols to determine basophil (identified with FcεRI-APC antibody, eBioscience) viability after culture.

### MHC Class II and costimulatory molecule expression

MHC Class II expression was detected on freshly isolated and stimulated basophils with combined FITC conjugated HLA-DR, -DP and -DQ antibodies and the corresponding IgG2a isotype-control antibody (BD Pharmingen). Costimulatory molecules were detected with anti-CD80-PE, anti-CD40-FITC, and anti-CD86-FITC (BD Pharmingen) and corresponding isotype control antibodies (IgG1, BD). Basophils were blocked with 5% human AB serum in PBS (FACS buffer) prior to antibody staining to inhibit non-specific Fc-IgG2a binding on the cell surface. Subsequent antibody and isotype control incubations were also performed in FACS buffer on ice for 20 minutes. The basophil population was identified as IgE^hi^ or FcεRI^hi^ and CD203c^+^, CD4^−^, CD19^−^ after strict exclusion of doublets and non-viable, 7AAD^+^ cells. RT-PCR was performed to confirm corresponding gene expression as described below.

### Gene expression analysis

Freshly isolated and stimulated basophils (FcεRI^hi^, CD203c^+^, 7AAD^−^, CD4^−^, CD19^−^ cells) were sorted (FACS Aria, BD) according to positive or negative MHC Class II expression directly into a 96-well skirted PCR-plate (Eppendorf, Hamburg, Germany) containing RT reaction mix. Flow cytometry sorted CD19^+^ B cells and CD123^+^IgE^low^ pDC from freshly isolated PBMC served as positive controls for MHC Class II related gene expression. The RT reaction mix consisted of 25 µg/ml Oligo dT18 (Geneworks, Thebarton, Australia), 1% Triton X (Sigma-Aldrich), 0.8 mM dNTP, 23 U RNase OUT Ribonuclease Inhibitor, 75 U SuperScript III Reverse Trascriptase, 8 mM DTT, 1x First Strand buffer and ultrapure distilled H_2_O (Invitrogen). The plate was then heated at 50°C for 50 minutes followed by 70°C for 15 minutes in a PCR machine to transcribe the mRNA to cDNA. PCR of the obtained cDNA was performed with the platinum pfx polymerase kit (Invitrogen) according to the manufacturer's protocol. Primers (Table 1) were designed to cross exon-exon boundaries using UCSC In-Silico PCR software and synthesized by GeneWorks (Thebarton, Australia). PCR was performed on Mastercycler (Eppendorf) as follows: 94°C for 5 minutes followed by 36 cycles of 94°C for 30 seconds, 58°C for 30 seconds and 68°C for 30 seconds. The PCR products were then visualized by ultraviolet illumination after electrophoresis in 1.5% agarose gels (Invitrogen) containing 0.5 µg/ml ethidium bromide (Sigma-Aldrich). CD80 primers were tested on cDNA of CD19^+^ cells sorted from 1 µg/ml CpG-stimulated PBMC as a positive control prior to use. Reverse Transcriptase negative controls of the CpG-stimulated CD19^+^ cell population were included to rule out amplification of genomic regions.

**Table 1:** Primer sequences used for RT-PCR.

Gene	Forward primer (5′-3′)	Reverse primer (5′-3′)
B2M	GAGGCTATCCAGCGTACTCCAAAG	GCTGCTTACATGTCTCGATCCCAC
CD40	TGCCAGCCAGGACAGAAACTG	CCAGGTCTTTGGTCTCACAGCTTG
CD80	GGG AAC ACC TGG CTG AAG TGA C	CAT CTT GGG GCA AAG CAG TAG G
CD86	CACAGGAATGATTCGCTCCAC	GGCTGAGGGTCCTCAAGCTCTATAG
HLA-DRα	TGGGACCATCTTCATCATCAAGG	GGGCATTCCAT AGCAGAGACAGAC
HLA-DMα	AGTTGGGGAAGCTGGGTTGG	CTGAGCCCAGTCAGCAAATTCG
CD74	GAAGCAGGAGCTGTCGGGAA	GGAAGCTTCATGCGCAGGTTC
Cathepsin S	GTGGTTGGCTATGGTGAT CTTAATG	AACTCCTGGCCTCAAGTTGATATGC
CD123	GAGCTTCAGATACAAAAGAGAATGCAGC	GGGTCTTTCATGTGAGGGATGC

### Generation of T cell lines

HDM-specific T cell lines were generated from freshly isolated PBMC of two atopic HDM-allergic donors and one non-atopic donor using 5,6-CFSE according to our previously described protocol [33]. Ara h 1 peptide (ALMLPHFN)-specific T cells were generated in a previous study according to the same protocol [34]. Antigen-specific responses of all T cell lines were shown to be HLA-DR restricted as described [33].

### T cell proliferation assays

Basophils were isolated and incubated at 37°C with IFN-γ (100 ng/ml), GM-CSF (100 ng/ml) and IL-3 (10 ng/ml) in 10% FCS RPMI for 72 hours to induce MHC Class II expression. For the HDM-specific T cell assays, HDM extract (10 µg/ml) or medium alone was also added to the basophil stimulation culture. Basophils were then washed and plated into a 96-well plate at 2×10^5^ cells/ml in complete medium (cRPMI) consisting of 2 mM L-glutamine, 100 IU/mL penicillin-streptomycin and 5% human AB serum in RPMI-1640 (Sigma-Aldrich). Previously generated autologous antigen-specific T cells were CFSE stained (0.1 µM) and added to each well at 2×10^5^ cells/ml cRPMI. Cells were incubated at 37°C with antigen (HDM extract or Ara h 1 peptide ALMLPHFN, 10 µg/ml) or cRPMI alone for a further 72 to 120 hours. Basophil-T cell co-cultures were then washed and stained with anti-FcεRI-APC, anti-CD3-eF450 (eBiosciences), anti-CD4-APC eF780, anti-CD25-PE (or the corresponding isotype control IgG1-PE) (BD Pharmingen) and 7AAD in FACS buffer for 20 minutes on ice. Proliferation was assessed by loss of CFSE fluorescence and increased CD25 expression and data presented as percentage of CD3/CD4 positive T cells proliferating. Gates were set according to isotype control and unstimulated T cell controls. As a positive control for antigen presentation, autologous CD14^+^ monocytes separated from PBMC by flow cytometry using anti-CD14-APC (BD Pharmingen) were irradiated (5000 rad) and incubated at T cell: monocyte ratios ranging from 1∶1 to 50∶1.

## Results

### Highly purified basophils require IL-3 for survival in culture

**Figure 1:**
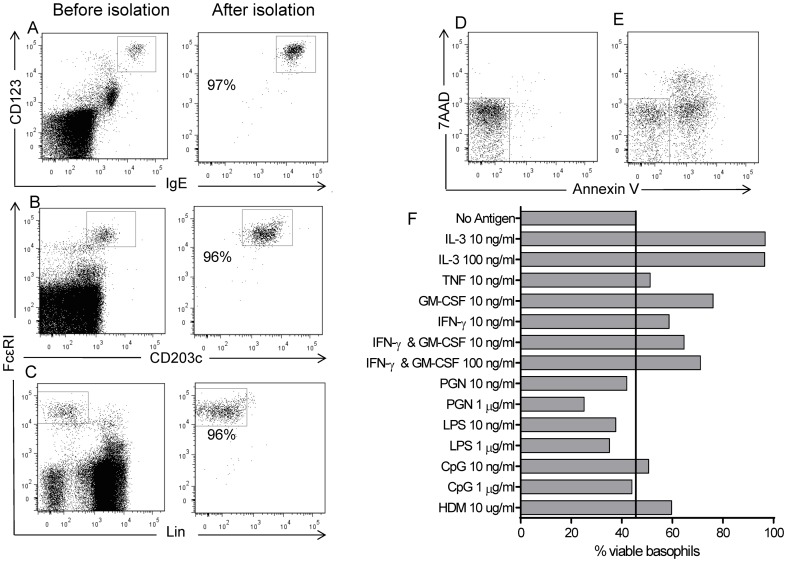
Isolated basophil purity and viability. Representative dot plots showing proportion of basophils in PBMC and after isolation assessed by antibodies specific for (A) IgE and CD123, (B) FcεRI and CD203c, (C) FcεRI and Lineage-1. Example of basophil viability after culture for 72 hours with (D) IL-3 (10 ng/ml) or (E) medium alone. (F) Percentage of viable basophils (7AAD^−^, Annexin V^−^) after 72 hours of culture with a panel of cytokines and TLR ligands. Representative of 2 separate experiments.

Freshly isolated basophils had high levels of surface bound IgE and expressed CD123 and FcεRI together with intermediate levels of the basophil-specific marker CD203c. They were CD4 and CD19 negative and, when included, lineage marker Lin-1 negative (Figure 1A, B, C). Purity levels of over 95% were consistently achieved as determined by expression of CD123 and high levels of surface bound IgE (Figure 1A). An additional pDC population expressing high levels of CD123, FcεRI, intermediate levels of CD4, but not CD203c was identified within freshly isolated PBMC. This population was excluded during the basophil isolation process.

The viability of isolated basophils was assessed in two separate experiments with the early apoptotic marker Annexin V and cell death marker 7AAD after 72 hours of culture with a panel of cytokines and TLR ligands. Less than half of the isolated basophil population were viable after culture for 72 hours in medium alone. Of the panel of stimuli tested, IL-3 was found to be most efficient at maintaining basophil viability for 72 hours of culture (Figure 1D, E, F). Therefore, 10 ng/ml of IL-3 was added to all cultures containing isolated basophils. Using IL-3 supplementation, viabilities of over 95% were obtained for isolated basophil preparations after 72 hours of culture throughout this study, as assessed by 7AAD.

### Basophils can be induced to express MHC Class II molecules

**Figure 2:**
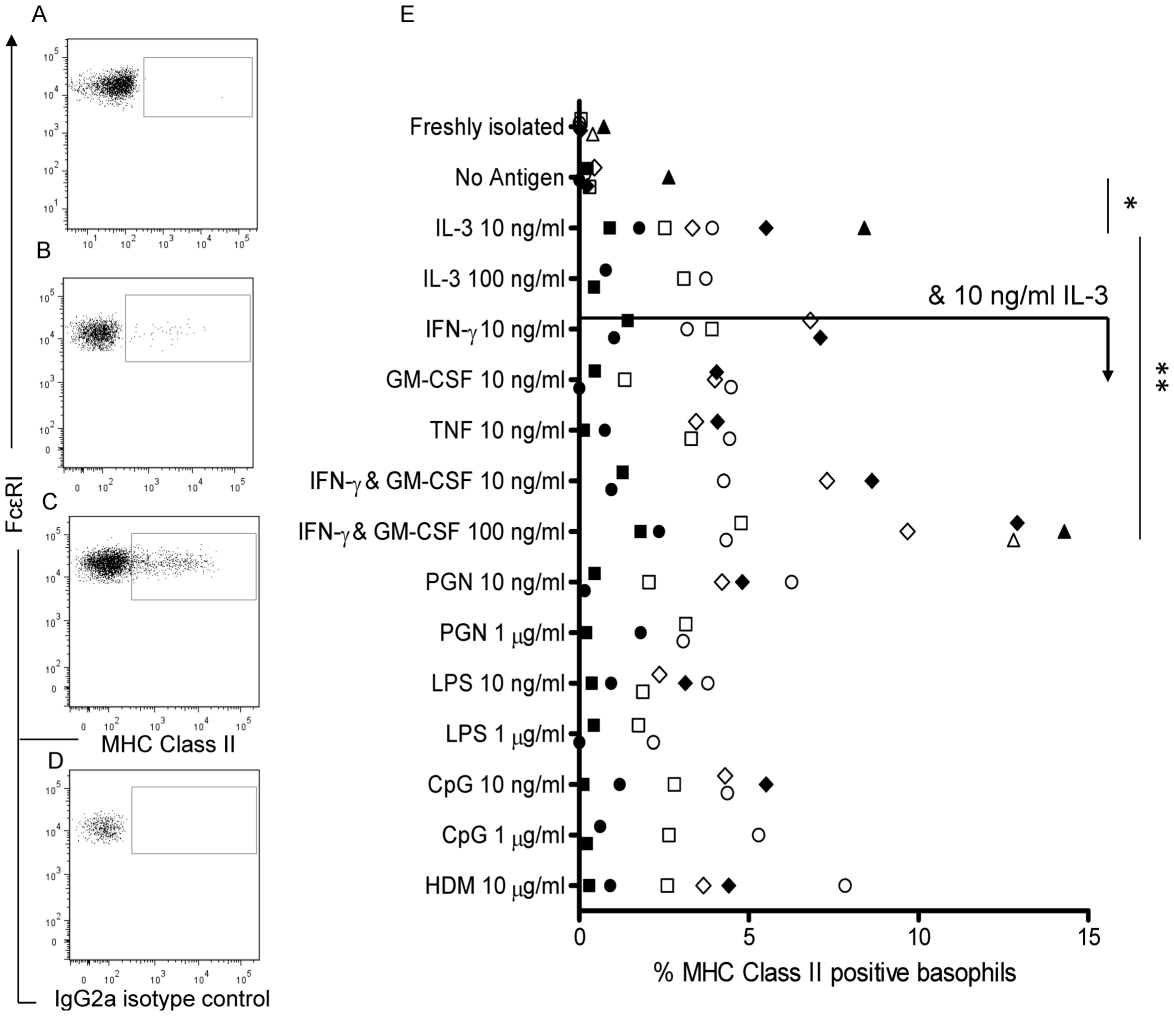
MHC Class II expression on stimulated basophils. Representative dot plots showing MHC Class II expression on basophils freshly isolated (A), and after 72 hours culture with medium alone (B) or IL-3 (10 ng/ml), IFN-γ and GM-CSF (100 ng/ml) (C). Corresponding isotype control (D). Percentage of MHC Class II positive basophils for individual atopic HDM-allergic (open shapes) and non-atopic (closed shapes) donors after 72 hours culture with various stimuli (E). Differences between groups were calculated with One-way ANOVA and Dunett's Multiple comparison post-test with IL-3 as the control group for complete data sets of the 6 donors, * p<0.05, ** p<0.01.

**Figure 3:**
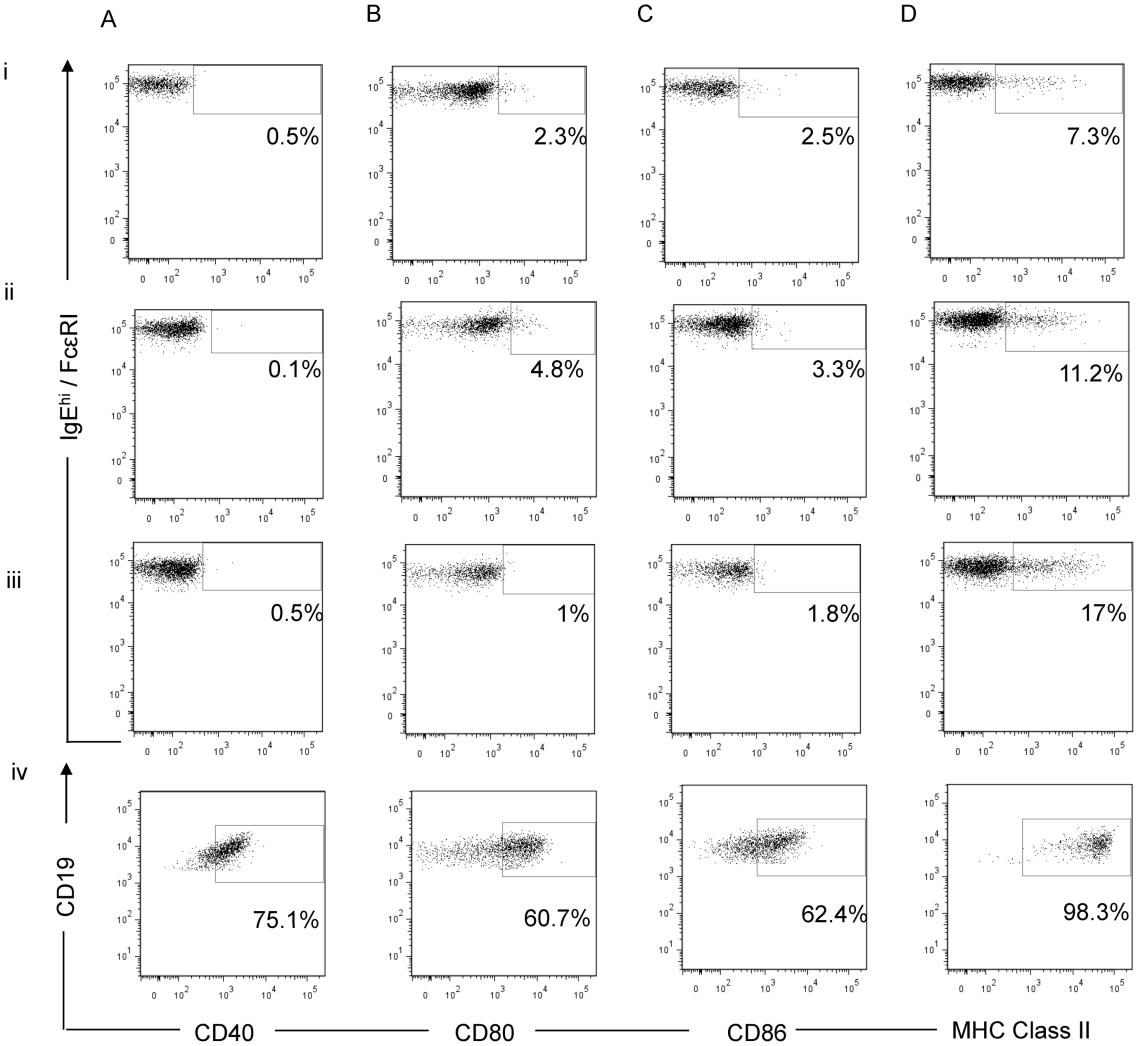
Costimulatory molecule expression on stimulated basophils. CD40 (A), CD80 (B), CD86 (C) and MHC Class II (D) expression on IL-3, IFN-γ and GM-CSF stimulated basophils of 2 atopic HDM-allergic (i,ii) and 1 non-atopic (iii) donor. CpG-stimulated B cells served as a positive control for costimulatory molecule detection (iv). Gates were determined using corresponding isotype controls.

We tested the induction of MHC Class II expression on circulating human basophils from 3 atopic HDM-allergic and 3 non-atopic donors with a panel of cytokines and TLR ligands selected on the basis of on known basophil receptors and previous eosinophil studies [26]–[30], [35]. We found that freshly isolated basophils did not express MHC Class II (Figure 2A, E), however a small but significant (p<0.05) increase in expression was observed when cells were cultured with IL-3 for 72 hours (Figure 2E). The addition of individual cytokines (IFN-γ, GM-CSF or TNF-α at 10 ng/ml), TLR ligands (PGN, LPS and CpG) or HDM allergen did not significantly increase this expression, but the addition of IFN-γ and GM-CSF (both at 100 ng/ml) combined, induced a higher percentage of MHC Class II positive basophils as compared to IL-3 alone (p<0.01) (Figure 2E). Two additional donors (1 atopic HDM-allergic and 1 non-atopic) were assessed for basophil MHC Class II expression upon stimulation with IL-3 (10 ng/ml), IFN-γ and GM-CSF (100 ng/ml) resulting in levels of 12.8 and 14.3% respectively, and in subsequent experiments the percentage of MHC Class II positive basophils in such culture conditions reached up to 17% (Figure 3D). The addition of HDM extract (10 µg/ml) to these cultures did not affect the percentage of MHC class II positive basophils (data not shown). The atopy status of subjects had no impact on basophil MHC Class II expression (Figure 2E).

### Costimulatory molecule expression on basophils remained minimal upon stimulation

Up-regulation of costimulatory molecules CD40, CD80 and CD86 was assessed by flow cytometry of basophils stimulated with a combination of IFN-γ, GM-CSF (100 ng/ml) and IL-3 (10 ng/ml) for three donors (two atopic HDM-allergic, one non-atopic) (Figure 3). Freshly isolated basophils did not express these costimulatory molecules. A small percentage of stimulated basophils (<5%) expressed CD80 and CD86 for both atopic donors however, this percentage was minimal compared to that for MHC Class II expressing cells. The addition of HDM extract (10 µg/ml) to these cultures did not increase costimulatory molecule expression (data not shown).

### Stimulated basophils contain RNA transcripts of MHC Class II components required for assembly and surface expression

**Figure 4:**
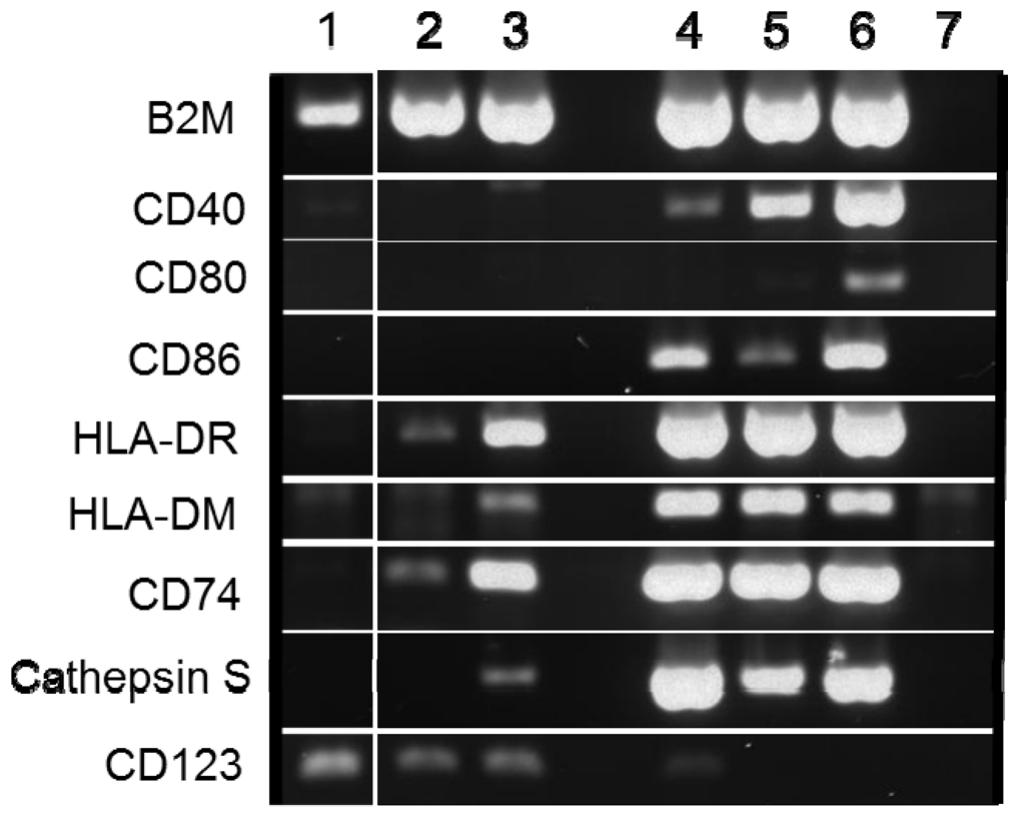
Gene expression of MHC Class II components and costimulatory molecules. RT-PCR of RNA from freshly isolated basophils (1), MHC Class II negative (2) and MHC Class II positive basophils (3) after 72 hours of culture with IL-3, IFN-γ, GM-CSF and HDM extract, compared with CD123^+^ IgE^low^ pDC (4), CD19^+^ B cells from freshly isolated PBMC (5) and CD19^+^ B cells from CpG stimulated PBMC (6) as positive controls, and a RT negative control (7). Data for a non-atopic donor are shown, representative of 5 separate experiments.

In order to confirm the flow cytometric detection of MHC Class II expression on isolated basophils after culture with IL-3, IFN-γ and GM-CSF and HDM extract, analysis of corresponding gene transcripts in sorted MHC Class II positive and negative basophils was performed with RT-PCR (representative data shown in Figure 4). The proteins HLA-DR, CD74, HLA-DM and Cathepsin S each play a role in achieving functional peptide-MHC Class II complex assembly on the cell surface. Expression levels of these genes were assessed with B2M used as the control housekeeping gene. HLA-DR, CD74 and Cathepsin S transcripts were detected at varying levels in MHC Class II positive basophils of all donors tested (3 atopic HDM-allergic, 2 non-atopic). HLA-DM transcripts were detected in MHC Class II positive basophils from 3 of the 5 donors. HLA-DR, CD74 and Cathepsin S transcripts were also detected (at decreased intensity levels) in stimulated basophils that were MHC Class II negative. Freshly isolated basophils did not contain any detectable MHC Class II related transcripts. Transcripts for CD40, CD80 or CD86 could not be detected in freshly isolated or stimulated basophils. Freshly isolated B cells and pDC expressed all transcripts of the MHC Class II related proteins as well as CD40 and CD86. CD80 transcripts were detected in CpG stimulated B cells.

### Basophils do not induce antigen-specific T cell proliferation

**Figure 5:**
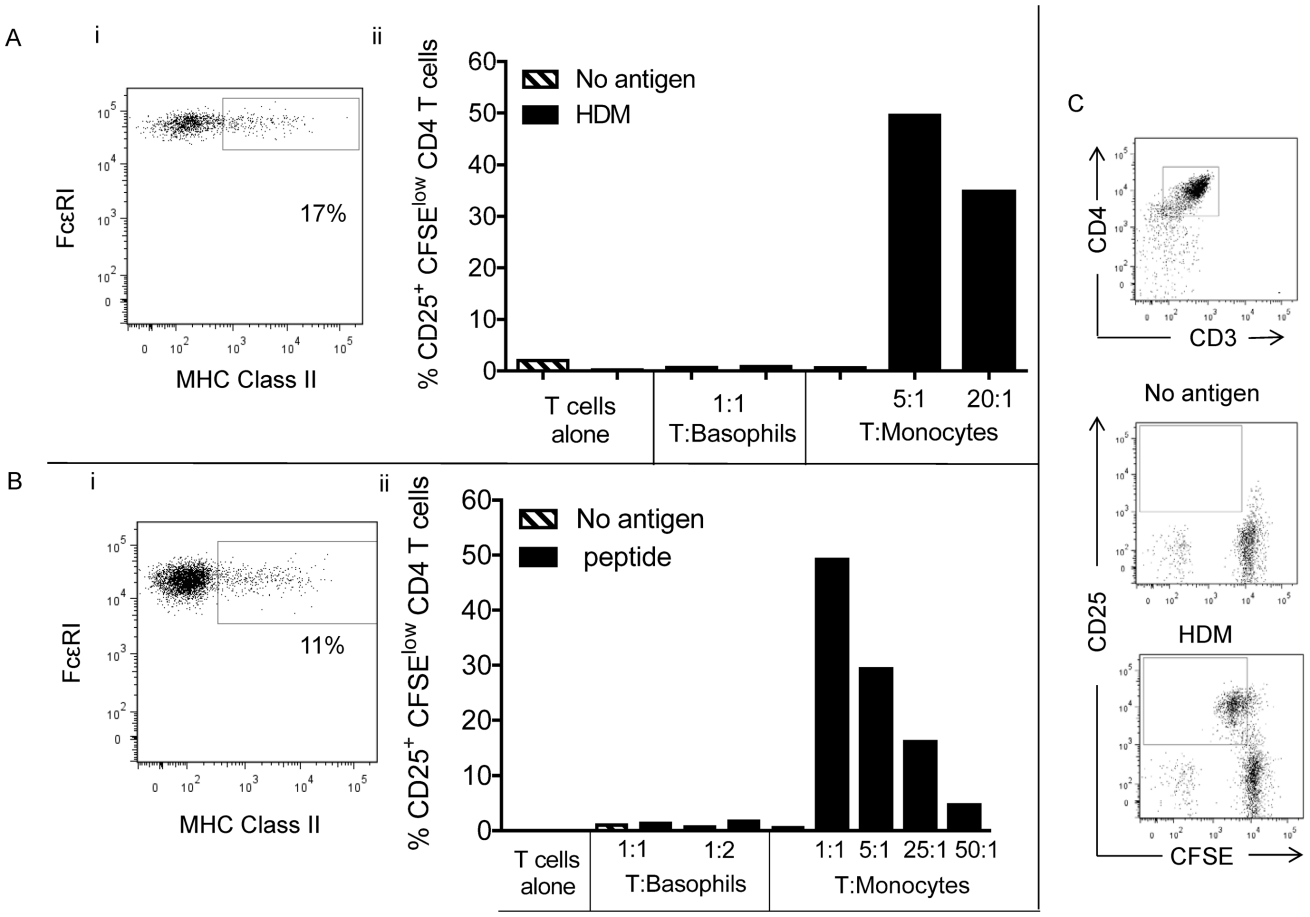
T cell proliferation assay. (i) Percentage of MHC Class II positive basophils after 72 hours culture with IL-3, IFN-γ and GM-CSF prior to co-culture with T cells. HDM extract (10 µg/ml) was also added to the basophil stimulation culture for A. (ii) Percentage of CD25^+^ CFSE^low^ proliferating CD3^+^CD4^+^ T cells after culture of T cells alone or with autologous basophils or monocytes in the presence of 10 µg/ml HDM (A, non-atopic donor; similar results for 2 HDM-allergic donors) for 72 hours or 10 µg/ml peptide (B) for 120 hours. T cell: APC ratio indicated below. (C) Representative dot plot showing gating strategy for identification of proliferating CD3^+^CD4^+^ T cells.

We next assessed the ability of stimulated human basophils to present antigen to established antigen-specific T cell lines and induce T cell proliferation. Isolated basophils were stimulated for 72 hours with IL-3, IFN-γ and GM-CSF (to induce MHC Class II expression) together with 10 µg/ml HDM or medium alone. Autologous CFSE-stained HDM-specific T cells were then added (1∶1 ratio) and the cells cultured in the presence of 10 µg/ml HDM or medium alone for an additional 72 hours. CD3^+^CD4^+^ 7AAD^−^ T cells were assessed by flow cytometry for activation and proliferation by up-regulation of CD25 expression and loss of CFSE fluorescence respectively. HDM-specific T cell lines from one non-atopic donor and two atopic HDM-allergic donors were tested (Figure 5A). No antigen-specific T cell proliferation was induced by stimulated basophils for any donor, indicating that although up to 17% of these cells expressed MHC Class II, they were incapable of antigen processing and/or presentation. As a positive control, flow cytometry-sorted monocytes were used to compare antigen presenting abilities of basophils with professional antigen presenting cells. This revealed that even at a 1∶20 monocyte to T cell ratio, these highly efficient antigen presenting cells were capable of inducing substantial antigen-specific T cell activation and proliferation, whereas basophils at a 1∶1 ratio with T cells were incapable of these tasks.

### Basophils do not present exogenous peptide to peptide-specific T cells

To investigate the possibility that the lack of T cell stimulatory activity was due to an inability to ingest or process antigen, we tested basophils for presentation of an exogenous peptide to a T cell line specific for that peptide. Basophils were stimulated with IL-3, IFN-γ and GM-CSF for 72 hours to induce MHC Class II expression, then washed and added to autologous CFSE-stained Ara h 1 peptide-specific T cells with 10 µg/ml peptide or medium alone (Figure 5B). Again, in contrast to monocytes, MHC Class II expressing basophils were incapable of inducing peptide-specific T cell activation/proliferation through peptide presentation, even after 5 days of culture. Furthermore, an increase in basophil to T cell ratio, from 1∶1 to 2∶1, did not improve their ability to induce proliferation.

## Discussion

The potential for basophils to present antigen to T cells has been of great interest in recent years. As basophils are capable of producing high levels of Th2 type cytokines and can be directly activated by cysteine proteases derived from both parasites and allergens [36], it was hypothesized that these cells could provide the answer to the initiation and development of a Th2 type T cell response. However, recent reports examining circulating human basophils have found these cells to be incapable of antigen presentation, showing lack of antigen uptake and insufficient MHC Class II and costimulatory molecule expression. We sought to induce these qualities in isolated human basophils through stimulation with a panel of cytokines, TLR ligands and HDM allergen for 72 hours. In this report we demonstrate for the first time that expression of essential components of MHC Class II can be induced in isolated human basophils with an appropriate cytokine mix, but expression of conventional costimulatory molecules including CD40, CD80 and CD86 is minimal. Despite the presence of surface MHC Class II, basophils were incapable of processing and presenting whole HDM allergen to HDM-specific T cells. Furthermore, basophils were unable to present exogenously added Ara h 1 peptide to induce proliferation of peptide-specific T cells.

Although some have previously reported a small percentage of basophils within freshly isolated PBMC to be MHC Class II positive [37]–[38], our findings agree with others [17]–[19] that show highly purified basophils enriched from freshly isolated PBMC are MHC Class II negative. Discrepancies between studies regarding the presence of MHC Class II molecules on unstimulated basophils may be due to the basophil isolation process or the detection technique used within whole PBMC. Our focus was on the ability to induce expression on mature human basophils, and we found up to 17% of basophils expressed MHC Class II on the cell surface following culture for 72 hours with IL-3, IFN-γ and GM-CSF. To our knowledge, this is the highest percentage of isolated mature human basophils induced to express MHC Class II reported to date. Furthermore, we are the first to show that the presence of surface bound MHC Class II was accompanied by expression of RNA transcripts of components required for assembly and surface expression of the complex, including HLA-DR, CD74, Cathepsin S and HLA-DM. Interestingly, MHC Class II-related transcripts were also found in surface MHC Class II negative basophils from the stimulated cultures indicating the potential for an even larger percentage of basophils to express the complex under appropriate conditions or upon prolonged stimulation.

In order to obtain a pure basophil population, we used a basophil enrichment kit based on negative selection that does not contain an HLA-DR-specific antibody. Furthermore, we applied strict exclusion of any doublets, FcεRI and CD203c negative cells, and CD19 and CD4 positive cells during FACS sorting for gene expression analysis. Since pDC of healthy individuals as well as basophils express CD123, we sorted CD123^+^IgE^low^ pDC and confirmed that, like the purified basophils, they expressed CD123 gene transcripts, but they were clearly distinguished from basophils by their high gene expression levels of all MHC Class II components as well as CD86 (Figure 4). Although measurable up-regulation of MHC Class II was detected in basophils after stimulation, similar studies involving eosinophils showed a much larger increase in the percentage of MHC Class II positive cells often reaching values of between 50 and 90% [26], [29]. In addition, expression of the costimulatory molecule CD86 has been detected on stimulated eosinophils [27]–[28]. Expression of conventional costimulatory molecules remained minimal in basophils of our donors upon stimulation as detected by both flow cytometry and RT-PCR.

Our T cell proliferation assays revealed that, despite the presence of MHC Class II molecules on the surface of stimulated basophils, these cells were incapable of processing and/or presenting T cell epitopes from whole antigen (HDM) to established T cell lines. In order to investigate this further, and remove the requirement for antigen processing, we also assessed the ability of stimulated basophils to present exogenous peptide to established T cell lines. Again, basophils were incapable of inducing peptide-specific T cell activation/proliferation, indicating additional functional deficiencies in antigen presentation distinct from an inability to process whole antigen.

The inability of basophils to induce peptide-specific T cell activation/proliferation may be due to insufficient costimulatory molecule expression, conventional or otherwise. Costimulatory molecules including ICAM-1 and LFA-3 [39] have been shown to assist in antigen presentation by eosinophils lacking conventional costimulatory molecules [31]. There are two reports supporting LFA-3 expression by human basophils [40]-[41], but the results from our peptide assay suggest that this costimulatory molecule expression (if present) together with MHC Class II is not sufficient to induce T cell activation/proliferation to peptide.

Although only 11–17% of basophils were found to express MHC Class II on their cell surface prior to T cell co-culture, by testing both a 1∶1 and 1∶2 T cell to basophil ratio in our assays, the actual ratio of T cells to MHC Class II positive basophils was within the range found to result in strong T cell response when stimulated by monocytes. In fact, monocytes could still induce high levels of T cell proliferation at much higher T cell to monocyte ratios than were employed for the basophils. In our T cell assays, we chose to include the entire basophil population to minimize manipulation of the cells. The flow cytometry-based method of assessing antigen presentation allows for highly sensitive detection of both early T cell activation (CD25 expression) and proliferation (CFSE loss), therefore even very low levels of T cell activation would have been detected. In conclusion, we demonstrate that mature human basophils can be induced to express MHC Class II, however this expression is insufficient to induce antigen-specific T-cell proliferation with either whole antigen or exogenous peptide.

## References

[1] Ohnmacht C, Voehringer D (2009) Basophil effector function and homeostasis during helminth infection. Blood 113: 2816–2825. 18941115 10.1182/blood-2008-05-154773

[2] Valent P, Bettelheim P (1990) The human basophil. Crit Rev Oncol Hematol 10: 327–352. 2278641 10.1016/1040-8428(90)90009-h

[3] Valent P, Bettelheim P (1992) Cell surface structures on human basophils and mast cells: biochemical and functional characterization. Adv Immunol 52: 333–423. 1332448 10.1016/s0065-2776(08)60879-2

[4] MacGlashan D Jr, White JM, Huang SK, Ono SJ, Schroeder JT, et al (1994) Secretion of IL-4 from human basophils. The relationship between IL-4 mRNA and protein in resting and stimulated basophils. J Immunol 152: 3006–3016. 8144899

[5] Schroeder JT, MacGlashan DW Jr, Kagey-Sobotka A, White JM, Lichtenstein LM (1994) IgE-dependent IL-4 secretion by human basophils. The relationship between cytokine production and histamine release in mixed leukocyte cultures. J Immunol 153: 1808–1817. 7519213

[6] Li H, Sim TC, Alam R (1996) IL-13 released by and localized in human basophils. J Immunol 156: 4833–4838. 8648131

[7] Ochensberger B, Daepp GC, Rihs S, Dahinden CA (1996) Human blood basophils produce interleukin-13 in response to IgE-receptor-dependent and -independent activation. Blood 88: 3028–3037. 8874201

[8] Gauchat JF, Henchoz S, Mazzei G, Aubry JP, Brunner T, et al (1993) Induction of human IgE synthesis in B cells by mast cells and basophils. Nature 365: 340–343. 7690905 10.1038/365340a0

[9] Yanagihara Y, Kajiwara K, Basaki Y, Ikizawa K, Ebisawa M, et al (1998) Cultured basophils but not cultured mast cells induce human IgE synthesis in B cells after immunologic stimulation. Clin Exp Immunol 111: 136–143. 9472673 10.1046/j.1365-2249.1998.00474.x PMC1904864

[10] Perrigoue JG, Saenz SA, Siracusa MC, Allenspach EJ, Taylor BC, et al (2009) MHC class II-dependent basophil-CD4+ T cell interactions promote T(H)2 cytokine-dependent immunity. Nat Immunol 10: 697–705. 19465906 10.1038/ni.1740 PMC2711559

[11] Sokol CL, Chu NQ, Yu S, Nish SA, Laufer TM, et al (2009) Basophils function as antigen-presenting cells for an allergen-induced T helper type 2 response. Nat Immunol 10: 713–720. 19465907 10.1038/ni.1738 PMC3252751

[12] Yoshimoto T, Yasuda K, Tanaka H, Nakahira M, Imai Y, et al (2009) Basophils contribute to T(H)2-IgE responses in vivo via IL-4 production and presentation of peptide-MHC class II complexes to CD4+ T cells. Nat Immunol 10: 706–712. 19465908 10.1038/ni.1737

[13] Hammad H, Plantinga M, Deswarte K, Pouliot P, Willart MA, et al (2010) Inflammatory dendritic cells—not basophils—are necessary and sufficient for induction of Th2 immunity to inhaled house dust mite allergen. J Exp Med 207: 2097–2111. 20819925 10.1084/jem.20101563 PMC2947072

[14] Kim S, Prout M, Ramshaw H, Lopez AF, LeGros G, et al (2010) Cutting edge: basophils are transiently recruited into the draining lymph nodes during helminth infection via IL-3, but infection-induced Th2 immunity can develop without basophil lymph node recruitment or IL-3. J Immunol 184: 1143–1147. 20038645 10.4049/jimmunol.0902447 PMC2849628

[15] Ohnmacht C, Schwartz C, Panzer M, Schiedewitz I, Naumann R, et al (2010) Basophils orchestrate chronic allergic dermatitis and protective immunity against helminths. Immunity 33: 364–374. 20817571 10.1016/j.immuni.2010.08.011

[16] Otsuka A, Nakajima S, Kubo M, Egawa G, Honda T, et al (2013) Basophils are required for the induction of Th2 immunity to haptens and peptide antigens. Nat Commun 4: 1738. 10.1038/ncomms2740 PMC3644090 23612279

[17] Eckl-Dorna J, Ellinger A, Blatt K, Ghanim V, Steiner I, et al (2012) Basophils are not the key antigen-presenting cells in allergic patients. Allergy 67: 601–608. 22335568 10.1111/j.1398-9995.2012.02792.x PMC4601523

[18] Kitzmuller C, Nagl B, Deifl S, Walterskirchen C, Jahn-Schmid B, et al (2012) Human blood basophils do not act as antigen-presenting cells for the major birch pollen allergen Bet v 1. Allergy 67: 593–600. 22188598 10.1111/j.1398-9995.2011.02764.x

[19] Sharma M, Hegde P, Aimanianda V, Beau R, Senechal H, et al (2013) Circulating human basophils lack the features of professional antigen presenting cells. Sci Rep 3: 1188. 23378919 10.1038/srep01188 PMC3561623

[20] Charles N, Hardwick D, Daugas E, Illei GG, Rivera J (2010) Basophils and the T helper 2 environment can promote the development of lupus nephritis. Nat Med 16: 701–707. 20512127 10.1038/nm.2159 PMC2909583

[21] Guo CB, Liu MC, Galli SJ, Bochner BS, Kagey-Sobotka A, et al (1994) Identification of IgE-bearing cells in the late-phase response to antigen in the lung as basophils. Am J Respir Cell Mol Biol 10: 384–390. 7510984 10.1165/ajrcmb.10.4.7510984

[22] Ito Y, Satoh T, Takayama K, Miyagishi C, Walls AF, et al (2011) Basophil recruitment and activation in inflammatory skin diseases. Allergy 66: 1107–1113. 21371044 10.1111/j.1398-9995.2011.02570.x

[23] Wakahara K, Baba N, Van VQ, Begin P, Rubio M, et al (2012) Human basophils interact with memory T cells to augment Th17 responses. Blood 120: 4761–4771. 23071273 10.1182/blood-2012-04-424226

[24] Arock M, Schneider E, Boissan M, Tricottet V, Dy M (2002) Differentiation of human basophils: an overview of recent advances and pending questions. J Leukoc Biol 71: 557–564. 11927641

[25] Grundstrom J, Reimer JM, Magnusson SE, Nilsson G, Wernersson S, et al (2012) Human cord blood derived immature basophils show dual characteristics, expressing both basophil and eosinophil associated proteins. PLoS One 7: e48308. 23118978 10.1371/journal.pone.0048308 PMC3485157

[26] Lucey DR, Nicholson-Weller A, Weller PF (1989) Mature human eosinophils have the capacity to express HLA-DR. Proc Natl Acad Sci U S A 86: 1348–1351. 2919183 10.1073/pnas.86.4.1348 PMC286687

[27] Jung YJ, Woo SY, Jang MH, Miyasaka M, Ryu KH, et al (2008) Human eosinophils show chemotaxis to lymphoid chemokines and exhibit antigen-presenting-cell-like properties upon stimulation with IFN-gamma, IL-3 and GM-CSF. Int Arch Allergy Immunol 146: 227–234. 18268391 10.1159/000115891

[28] Celestin J, Rotschke O, Falk K, Ramesh N, Jabara H, et al (2001) IL-3 induces B7.2 (CD86) expression and costimulatory activity in human eosinophils. J Immunol 167: 6097–6104. 11714768 10.4049/jimmunol.167.11.6097

[29] Weller PF, Rand TH, Barrett T, Elovic A, Wong DT, et al (1993) Accessory cell function of human eosinophils. HLA-DR-dependent, MHC-restricted antigen-presentation and IL-1 alpha expression. J Immunol 150: 2554–2562. 8450230

[30] Mawhorter SD, Kazura JW, Boom WH (1994) Human eosinophils as antigen-presenting cells: relative efficiency for superantigen- and antigen-induced CD4+ T-cell proliferation. Immunology 81: 584–591. 7518797 PMC1422364

[31] Hansel TT, De Vries IJ, Carballido JM, Braun RK, Carballido-Perrig N, et al (1992) Induction and function of eosinophil intercellular adhesion molecule-1 and HLA-DR. J Immunol 149: 2130–2136. 1355503

[32] Eusebius NP, Papalia L, Suphioglu C, McLellan SC, Varney M, et al (2002) Oligoclonal analysis of the atopic T cell response to the group 1 allergen of Cynodon dactylon (bermuda grass) pollen: pre- and post-allergen-specific immunotherapy. Int Arch Allergy Immunol 127: 234–244. 11979049 10.1159/000053868

[33] Prickett SR, Voskamp AL, Dacumos-Hill A, Symons K, Rolland JM, et al. (2011) Ara h 2 peptides containing dominant CD4+ T-cell epitopes: candidates for a peanut allergy therapeutic. J Allergy Clin Immunol 127: :608–615 e601–605. 10.1016/j.jaci.2010.09.027 21093025

[34] Prickett SR, Voskamp AL, Phan T, Dacumos-Hill A, Mannering S, et al (2013) Ara h 1 CD4+ T-cell epitope-based peptides: candidates for a peanut allergy therapeutic. Clin Exp Allergy 43: 684–697. 23711131 10.1111/cea.12113 PMC3709139

[35] Komiya A, Nagase H, Okugawa S, Ota Y, Suzukawa M, et al (2006) Expression and function of toll-like receptors in human basophils. Int Arch Allergy Immunol 140 Suppl 123–27. 16772723 10.1159/000092707

[36] Phillips C, Coward WR, Pritchard DI, Hewitt CR (2003) Basophils express a type 2 cytokine profile on exposure to proteases from helminths and house dust mites. J Leukoc Biol 73: 165–171. 12525574 10.1189/jlb.0702356

[37] Poulsen BC, Poulsen LK, Jensen BM (2012) Detection of MHC class II expression on human basophils is dependent on antibody specificity but independent of atopic disposition. J Immunol Methods 381: 66–69. 22546486 10.1016/j.jim.2012.04.009

[38] Siegmund R, Vogelsang H, Machnik A, Herrmann D (2000) Surface membrane antigen alteration on blood basophils in patients with Hymenoptera venom allergy under immunotherapy. J Allergy Clin Immunol 106: 1190–1195. 11112905 10.1067/mai.2000.110928

[39] Damle NK, Klussman K, Linsley PS, Aruffo A (1992) Differential costimulatory effects of adhesion molecules B7, ICAM-1, LFA-3, and VCAM-1 on resting and antigen-primed CD4+ T lymphocytes. J Immunol 148: 1985–1992. 1372018

[40] Fureder W, Agis H, Sperr WR, Lechner K, Valent P (1994) The surface membrane antigen phenotype of human blood basophils. Allergy 49: 861–865. 7709996 10.1111/j.1398-9995.1994.tb00788.x

[41] Munitz A, Bachelet I, Fraenkel S, Katz G, Mandelboim O, et al (2005) 2B4 (CD244) is expressed and functional on human eosinophils. J Immunol 174: 110–118. 15611233 10.4049/jimmunol.174.1.110

